# Efficacy and safety of laser peripheral iridoplasty with different energy levels and locations in the treatment of primary angle closure disease assessed by swept-source anterior segment optical coherence tomography

**DOI:** 10.1186/s12886-023-02899-0

**Published:** 2023-04-11

**Authors:** Jiayin Qin, Yan Zhang, Chengxia Zhang, Yaqian Niu, Fei Yang, Xijuan Wang, Xiaochun Li, Yang Yu

**Affiliations:** Department of Ophthalmology, Peking University International Hospital, Life Park Road No. 1, Life Science Park of Zhong Guancun, Changping District, Beijing, 102206 China

**Keywords:** Laser peripheral iridoplasty, Laser energy, Laser location, Swept-source AS-OCT, Efficacy, Safety

## Abstract

**Background:**

To explore the efficacy and safety of laser peripheral iridoplasty (LPIp) with different energy levels and locations in the treatment of primary angle closure disease (PACD) assessed by swept-source anterior segment optical coherence tomography (AS-OCT).

**Methods:**

We enrolled patients with PACD following best-corrected visual acuity (BCVA), intraocular pressure (IOP), anterior chamber gonioscopy, ultrasound biomicroscopy(UBM), optic disc OCT, and visual field examinations. After Pentacam and AS-OCT measurements, the patients were randomly divided into four treatment groups for LPIp with two different energy levels (high vs. low energy) and two locations (far from the periphery vs. near the periphery) and combined with laser peripheral iridotomy. BCVA, IOP, pupil diameter, central anterior chamber depth, anterior chamber volume, anterior opening distance (AOD)500, AOD750, trabecular iris angle (TIA)500, and TIA750 in four quadrants before and after laser treatment were compared.

**Results:**

We followed up 32 patients (64 eyes; average age, 61.80 ± 9.79 years; 8 patients/16 eyes per group) for up to 2 years. The IOP of all enrolled patients was decreased after surgery compared to that before (t = 3.297, *P* = 0.002), the volume of the anterior chamber was increased (t=-2.047, *P* = 0.047), and AOD500, AOD750, TIA500, and TIA750 were increased (all *P* < 0.05). Within-group comparisons showed that BCVA in the low-energy/far-periphery group was improved after surgery (*P* < 0.05). After surgery, the IOP was decreased in the two high-energy groups, whereas the volume of the anterior chamber, AOD500, AOD750, TIA500, and TIA750 were increased in all groups (all *P* < 0.05). However, when comparing every two groups, the high-energy/far-periphery group showed a stronger effect on pupil dilation than the low-energy/near-periphery group (*P* = 0.045). The anterior chamber volume in the high-energy/near-periphery group was larger than that in the high-energy/far-periphery group (*P* = 0.038). The change in TIA500 was for 6 points smaller in the low-energy/near-periphery group than in the low-energy/far-periphery group (*P* = 0.038). Other parameters showed no significant group differences.

**Conclusion:**

LPIp combined with iridotomy can effectively reduce IOP, increase anterior chamber volume, increase chamber angle opening distance, and widen the trabecular iris angle. Intraoperatively, high-energy laser spots positioned one spot diameter from the scleral spur can obtain the best effect and safety. Swept-source AS-OCT can safely and effectively quantify the anterior chamber angle.

## Background

Primary angle closure glaucoma (PACG) is the leading cause of irreversible blindness worldwide [1]. Demographic studies confirm that the PACG prevalence in Asia is much higher than that in Europe and America [2, 3]. The pathophysiological characteristics and pathogenesis of primary angle closure (PAC) and PACG are complex. The classic pathogenesis of PAC is a pupillary block which can be relieved by laser peripheral iridotomy (LPI) to facilitate aqueous humour flow directly from the posterior to the anterior chamber. However, studies have shown that 19.4–42.9% of patients with PAC still have a closed angle after LPI [4]. This confirms that mechanisms other than a pupillary block can cause PAC, including plateau iris, hypertrophic peripheral iris, forward position of the lens, and increased lens thickness [5]. These are not only independent mechanisms of angle closure but also coexist with pupillary block mechanisms. For patients with angle closure caused by lens factors, usually lens extraction is the treatment of choice. For patients with plateau or hypertrophic iris, laser peripheral iridoplasty (LPIp) combined with LPI is the preferred treatment.

LPIp is a safe and simple operation, which can effectively open the closed angle [6]. At present, different spot locations, spot numbers, and energy levels are used according to the personal experience and preference of the ophthalmologist. So far, little research has been conducted regarding the impact of laser photocoagulation location and energy level on treatment efficacy and safety. The presumed LPIp mechanism suggests that the closer the laser spot is to the periphery, the stronger the increase in the angle altered by iris atrophy, the weaker the side effect of pupil dilation, and the greater the risk of causing laser-induced peripheral anterior synechiae (PAS).

Traditional AS-OCT uses a single cross-sectional scan, whereas swept-source AS-OCT is a new technology for anterior segment imaging. It uses a scanning laser that can quickly capture multiple images. An accurate evaluation of the angle helps to identify patients with suspected primary angle closure (PACS).

In this study, we used swept-source AS-OCT to study the efficacy and safety of LPIp with different energies and locations in PAC treatment.

## Materials and methods

### Study population

We enrolled patients with PACS and PAC who visited the Ophthalmology Department of Peking University International Hospital from December 2019 to December 2021. In order to avoid the bias of lost visits, we included patients who had lived locally for a long time, and reminded them to come for further consultation by telephone. To avoid information deviation, each examination is completed by the same examiner, and all laser operations are completed by the same doctor (Dr Qin).

The inclusion criteria of patients were as follows: (1) Newly diagnosed patients with PACS or PAC, (2) an IOP of less than 30 mmHg, and (3) UBM showed a plateaued or hypertrophic peripheral iris. PACS was defined as a narrow chamber angle (posterior pigmented trabecular meshwork not visible in at least three quadrants during static gonioscopy), an IOP of less than 21 mmHg, no glaucoma-induced optic nerve injury, and no peripheral anterior adhesion. PAC was defined as the presence of a closed angle (peripheral iris and posterior trabecular meshwork not visualized in more than 180 degrees), elevated IOP, and PAS without glaucoma-induced optic disc or visual field damage [7, 8].

The exclusion criteria were as follows: (1) a baseline IOP without treatment above 30 mmHg, (2) systemic diseases or poor fixation, making cooperation of the patient with the examination and laser treatment impossible, (3) other diseases in the anterior or posterior segment of the eye or prior eye surgery, (4) systemic or local use of drugs that affect pupil diameter or the physiological IOP rhythm, and (5) secondary angle closure (e.g., neovascularization, uveitis, retinitis pigmentosa).

Potential patients were included based on the results of the BCVA, IOP, anterior chamber gonioscopy (OSMG, ocular), UBM (Aviso, Quantel Medical), and visual field (OCTOPUS900, Clinico) examinations.

### AS-OCT measurements

**Fig. 1:**
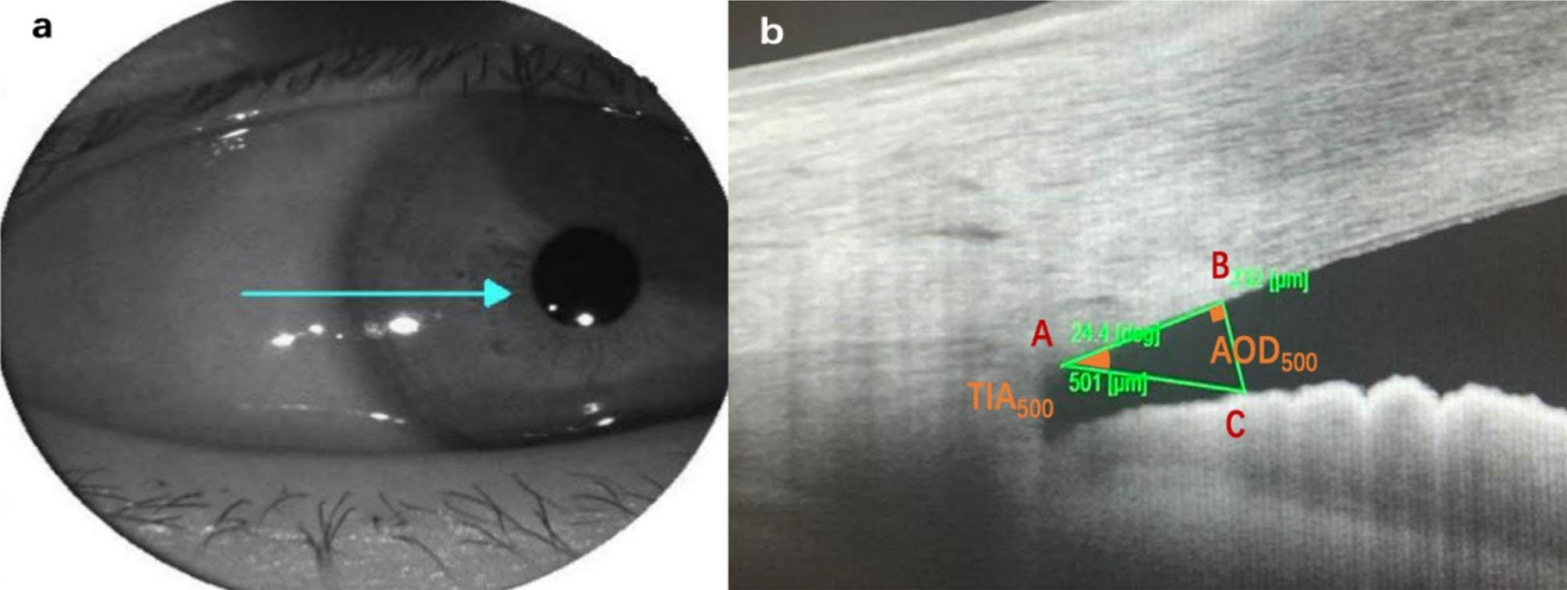
Method of measuring parameters of the anterior chamber angle based on DRI Triton (Topcon, Tokyo, Japan) swept anterior segment optical coherence tomography. AOD: anterior opening distance;  TIA: trabecular iris angle

Before and after laser therapy, all enrolled patients were examined in a dark room with swept-source AS-OCT (DRI Triton, Topcon) to measure anterior chamber angle parameters. The sitting patient placed the head onto the chinrest and adjusted the eye position. During the examination, the patient’s eyelids were gently pulled to expose the corneal limbus. The linear scanning mode of the AS-OCT (wavelength 1050 nm) was used to obtain images of the anterior chamber angle at 12, 6, 3, and 9 o’clock. The scanning range was 6 mm centred on the limbus of the cornea with 64 superimpositions. Only pictures that clearly showed the scleral process were selected for analysis. The built-in ImageJ software (National Institutes of Health, Bethesda, MD, USA) was used for image processing to measure the AOD 500/750 and TIA500/750. First, the scleral spur, which is the point where the curvature of the corneoscleral interface changes, was manually identified as point A (Fig. 1). Then, a 500-µm long line from the scleral process that intersects the inner surface of the cornea at a point (B) was drawn, as well as a perpendicular line that crosses the front surface of the iris at another point (C). The length of B-C was defined as AOD500. Draw AOD750 in the same way. The intersection of AOD500/750 and the anterior surface of the iris forms a line with the scleral spur. The included angle of these two lines passing through the scleral spur was defined as TIA500/750, respectively.

### LPIp and LPI procedures

The enrolled patients were treated with LPIp combined with LPI after AS-OCT and Pentacam (70700, Oculus) scanning. One hour before the laser treatment, 2% pilocarpine nitrate eye drops (Bausch + Lomb, USA) and tobramycin dexamethasone eye drops (Alcon, USA) were alternately applied three times per eye drop with an interval of 5 min. Obucaine hydrochloride eye drops (Santen, Japan) were used for surface anaesthesia before treatment. First, LPI was carried out at the recess or the thinnest part of the iris, while avoiding the palpebral fissure area, using an Nd:YAG laser (Selecta Duet, LUMENIS) beam of about 0.2 mm in size. Then, LPIp was performed using an argon multi-wavelength fundus laser (Vision One, LUMENIS). Fixed parameters were a spot area of 500 μm and an exposure time of 0.7 s. The laser range comprised 16 points distributed over 360 degrees. Laser energy and spot location were variable parameters. Two levels of laser energy (high energy, dark grey indicating obvious atrophy; low energy, light grey indicating light atrophy) and two types of spot positions (far from the periphery, 2 spot diameters from the scleral spur; near the periphery, 1 spot diameter from the scleral spur) were randomly assigned to each patient through random envelope method, dividing the study population into four treatment groups. Group HEFP: high energy, far from the periphery; group HENP: high energy, near the periphery; group LEFP: low energy, far from the periphery; and group LENP: low energy, near the periphery. Postoperative medication included tobramycin dexamethasone eye drops (4 times/day for 1 week) and 2% pilocarpine (once per night for 3 weeks).

### Statistical analysis

The data of the measured parameters in this study showed a normal distribution in the W-test and are expressed in the form of mean ± standard deviation. Because the two eyes of each participant are in the same treatment group, we used the average value of two eyes for statistical comparison to avoid the need for adjusting for inter-eye correlation. The BCVA, IOP, pupil diameter, central anterior chamber depth, anterior chamber volume, AOD500, AOD750, TIA500, and TIA750 before and after laser treatment were tested using the paired t-test. The parameter changes in each group after laser treatment were compared between and within groups using one-way ANOVA. *P* < 0.05 was considered to indicate significant differences. IBM SPSS 24 software was used for all statistical analyses.

## Results

The 32 patients (64 eyes) enrolled in this study included 2 men and 30 women with an average age of 61.80 ± 9.79 (range, 42–77) years. The time of the last follow-up ranged from 6 months to 2 years. Each of the four study groups comprised 8 patients (16 eyes). The baseline values of pupil diameter and anterior chamber volume significantly differed among the four groups (*P* < 0.05), mainly because the pupil diameters of group HENP were larger than those of group HEFP and LEFP, and the anterior chamber volumes of group HENP were higher than those of group LENP. Age, BCVA, IOP, central anterior chamber depth, as well as AOD500, AOD750, TIA500, and TIA750 at each time point were not significantly different (Table 1).

**Table 1:** Patient characteristics

Variable		HEFP	HENP	LEFP	LENP	F	*P*
Number		16	16	16	16	-	-
Age (years)		65.50 ± 5.42	58.40 ± 6.62	63.60 ± 11.15	63.70 ± 8.00	1.98	0.127
Female/male sex		14/2	16/0	14/2	16/0	-	-
Time(days)		420.00	390.00	390.00	420	1.763	0.164
BCVA (logMAR)		0.22 ± 0.21	0.11 ± 0.19	0.30 ± 0.19	0.15 ± 0.15	2.671	0.056
IOP (mmHg)		17.06 ± 4.16	17.37 ± 1.86	19.70 ± 7.46	17.25 ± 5.95	0.553	0.650
Treatment energy(mW)		177.14 ± 10.69	184.00 ± 10.75	120.91 ± 6.84	116.25 ± 7.19	249.67	0.000
Pupil diameter (mm)		2.39 ± 0.28	2.94 ± 0.31	2.68 ± 0.36	2.92 ± 0.50	4.871	0.006
Central anterior chamber depth (mm)		2.00 ± 0.15	2.00 ± 0.18	2.12 ± 0.19	1.90 ± 0.29	1.981	0.134
Anterior chamber volume (mm^3^)		71.20 ± 7.47	80.00 ± 12.10	88.90 ± 10.29	76.80 ± 20.16	3.069	0.040
AOD500 (µm)	12:00	72.70 ± 56.99	67.00 ± 76.43	110.10 ± 87.19	95.11 ± 94.74	0.624	0.604
	6:00	84.60 ± 84.48	134.30 ± 69.67	124.90 ± 67.87	69.70 ± 67.86	1.832	0.159
	9:00	187.30 ± 58.36	146.80 ± 65.86	164.20 ± 86.28	108.00 ± 82.83	2.030	0.127
	3:00	146.90 ± 57.82	171.10 ± 67.64	156.89 ± 66.11	109.90 ± 75.74	1.508	0.229
AOD750 (µm)	12:00	113.50 ± 44.49	101.20 ± 87.86	164.80 ± 122.71	123.00 ± 112.65	0.814	0.495
	6:00	136.00 ± 69.83	166.70 ± 86.31	164.10 ± 75.23	109.00 ± 98.68	1.062	0.377
	9:00	228.20 ± 43.72	194.00 ± 64.83	238.10 ± 98.00	160.60 ± 119.79	1.654	0.194
	3:00	235.50 ± 191.43	205.20 ± 89.49	223.78 ± 62.75	145.00 ± 120.26	0.995	0.406
TIA500 (degree)	12:00	8.32 ± 6.48	7.66 ± 8.59	12.60 ± 9.71	10.46 ± 10.30	0.634	0.598
	6:00	10.03 ± 9.49	14.94 ± 7.19	14.60 ± 7.09	8.06 ± 7.99	1.813	0.162
	9:00	20.41 ± 5.70	16.39 ± 6.37	17.50 ± 8.03	11.96 ± 9.35	2.186	0.107
	3:00	16.53 ± 6.17	18.29 ± 7.01	17.33 ± 6.08	12.42 ± 8.07	1.393	0.261
TIA750 (degree)	12:00	8.38 ± 3.16	7.64 ± 6.57	12.20 ± 8.55	9.35 ± 7.83	0.852	0.474
	6:00	10.13 ± 5.04	12.64 ± 6.41	13.00 ± 5.75	8.83 ± 7.96	0.987	0.410
	9:00	16.88 ± 3.13	14.51 ± 4.14	17.90 ± 6.71	11.77 ± 8.44	2.080	0.120
	3:00	14.19 ± 6.29	15.32 ± 6.00	16.89 ± 4.37	11.30 ± 8.45	1.457	0.243

AOD: anterior opening distance; BCVA: best-corrected visual acuity; IOP: intraocular pressure; TIA: trabecular iris angle; HEFP: high energy, far from the periphery; HENP: high energy, near the periphery; LEFP: low energy, far from the periphery; LENP: low energy, near the periphery

**Fig. 2:**
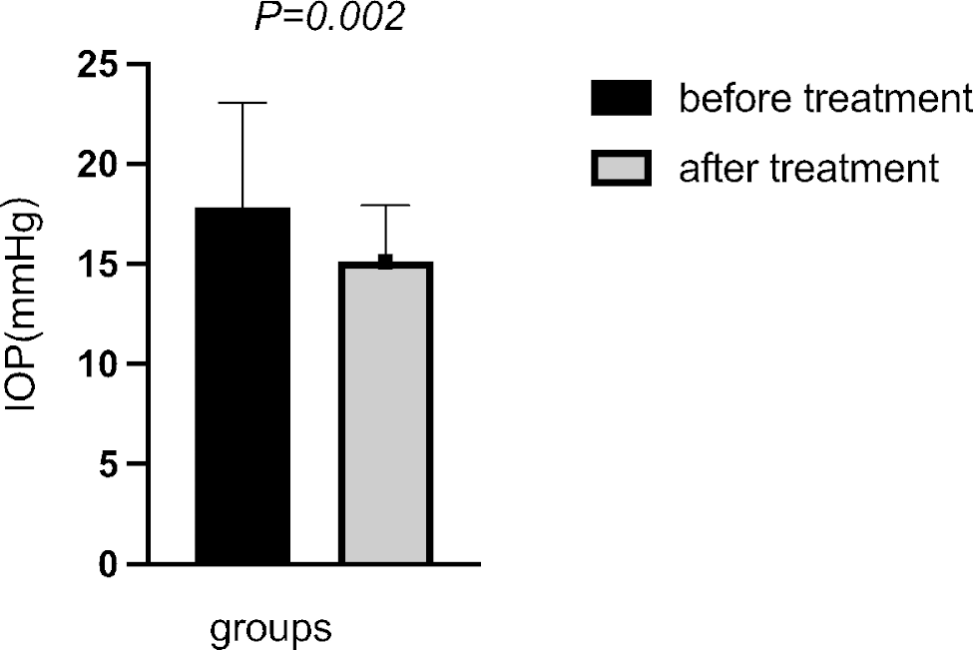
Comparison of the IOP before and after treatment

**Fig. 3:**
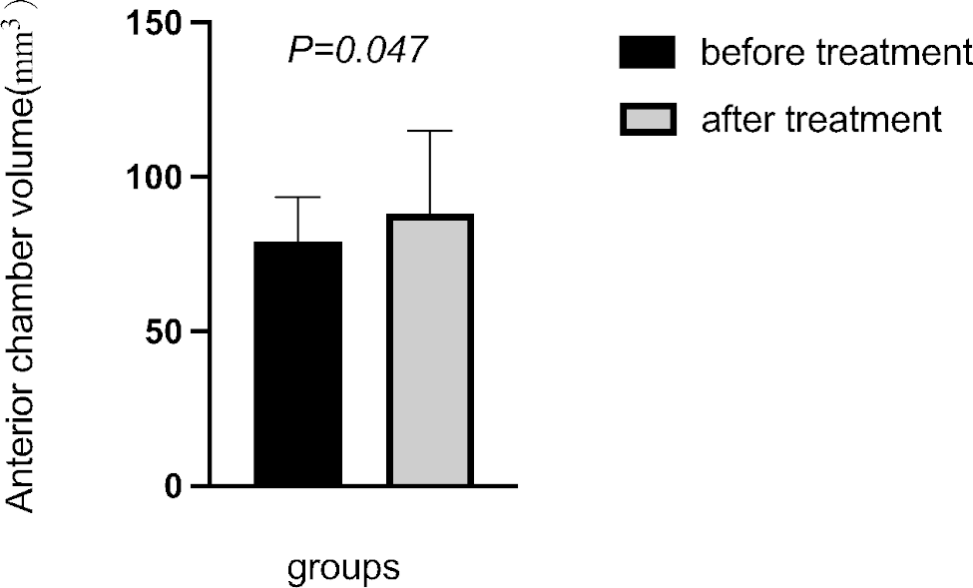
Comparison of the anterior chamber volume before and after treatment

**Fig. 4:**
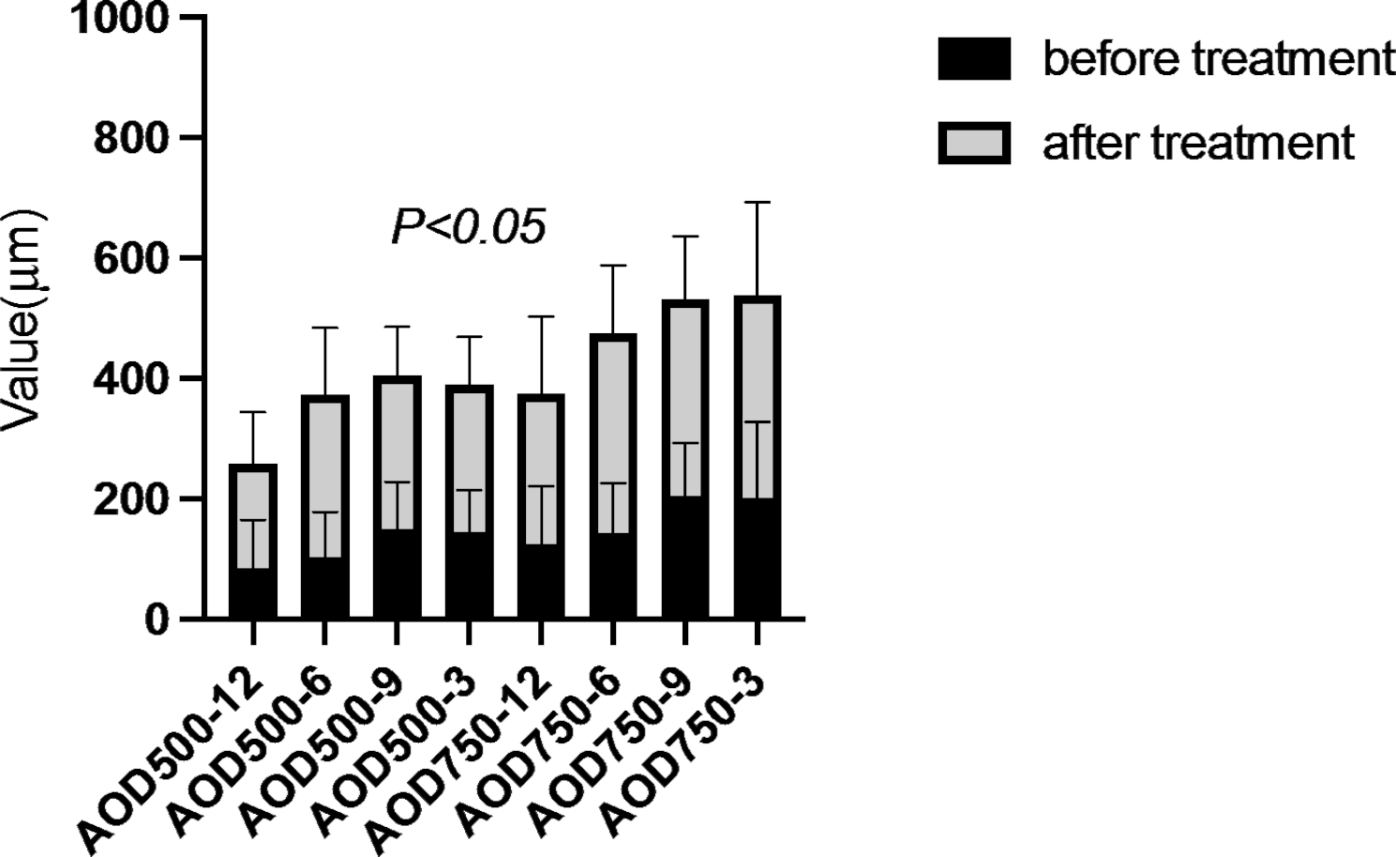
Comparison of the anterior opening distance (AOD) before and after treatment

**Fig. 5:**
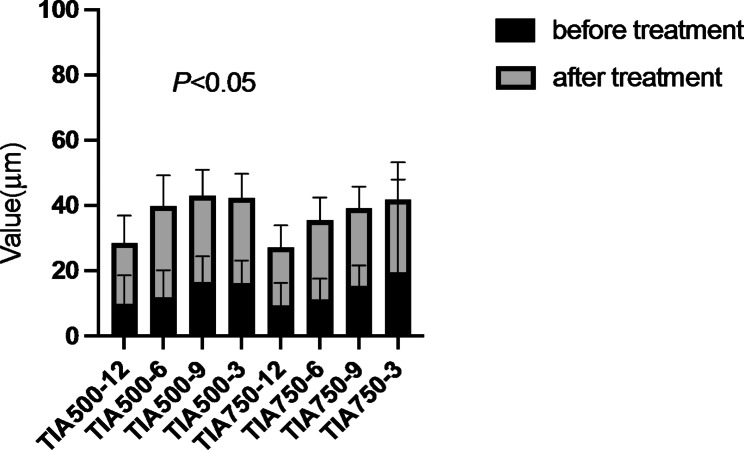
Comparison of the trabecular iris angles (TIAs) before and after treatment

BCVA, IOP, pupil diameter, central anterior chamber depth, anterior chamber volume, AOD500, AOD750, TIA500, and TIA750 of all enrolled patients before and after surgery were compared. The IOP after surgery was significantly lower than that before (t = 3.297, *P* = 0.002). The anterior chamber volume after surgery was significantly higher than that before (t=-2.047, *P* = 0.047). AOD500, AOD750, TIA500, and TIA750 were significantly increased after surgery compared to the values before the intervention (all *P* < 0.05). However, no significant differences in BCVA, pupil diameter, and central anterior chamber depth were found (Table 2; Figs. 2, 3, 4 and 5).

**Table 2:** Comparison of therapeutic effects before and after surgery

Variable		Before surgery	After surgery	t	*P*
BCVA (logMAR)		0.18 ± 0.19	0.16 ± 0.16	0.774	0.444
IOP (mmHg)		17.85 ± 5.20	15.12 ± 2.80	3.297	0.002
Pupil diameter (mm)		2.73 ± 0.42	4.15 ± 6.10	-1.499	0.142
Central anterior chamber depth (mm)		2.00 ± 0.22	2.05 ± 0.35	-1.129	0.266
Anterior chamber volume (mm^3^)		79.23 ± 14.39	88.05 ± 26.90	-2.047	0.047
AOD500 (µm)	12:00	86.23 ± 78.94	172.65 ± 85.63	-7.992	0.000
	6:00	103.35 ± 75.10	270.03 ± 110.88	-8.875	0.000
	9:00	151.58 ± 77.13	253.88 ± 81.18	-8.462	0.000
	3:00	145.92 ± 68.49	244.26 ± 79.55	-8.485	0.000
AOD750 (µm)	12:00	125.63 ± 96.06	249.68 ± 128.78	-6.227	0.000
	6:00	143.95 ± 83.45	331.70 ± 112.68	-9.980	0.000
	9:00	205.23 ± 88.82	327.78 ± 103.29	-8.226	0.000
	3:00	201.82 ± 126.87	337.05 ± 153.64	-5.068	0.000
TIA500 (degree)	12:00	9.76 ± 8.77	18.72 ± 8.36	-8.493	0.000
	6:00	11.91 ± 8.24	27.96 ± 9.30	-9.261	0.000
	9:00	16.57 ± 7.83	26.37 ± 8.04	-8.952	0.000
	3:00	16.11 ± 7.01	26.28 ± 7.29	-9.146	0.000
TIA750 (degree)	12:00	9.39 ± 6.81	17.70 ± 6.77	-8.817	0.000
	6:00	11.15 ± 6.38	24.32 ± 6.94	-10.546	0.000
	9:00	15.27 ± 6.23	23.91 ± 6.63	-8.641	0.000
	3:00	14.19 ± 6.29	22.05 ± 6.20	-8.187	0.000

AOD: anterior opening distance; BCVA: best-corrected visual acuity; IOP: intraocular pressure; TIA: trabecular iris angle

The parameters before and after surgery were also compared within each group. The BCVA of group LEFP was significantly improved after surgery, whereas no significant differences before and after surgery were found in any of the other groups. The IOP values of HEFP and HENP were significantly decreased after surgery compared to those before surgery (both *P* < 0.05). In the other two groups, no statistically significant differences before and after surgery were found. Moreover, no significant differences in pupil diameter and central anterior chamber depth before and after surgery were found in any of the study groups. In all groups, the anterior chamber volume, AOD500, AOD750, TIA500, and TIA750 were significantly increased after surgery compared to the value before surgery (all *P* < 0.05; Table 3).

**Table 3:** Comparison of therapeutic effects among groups

Variable		HEFP (n = 16)			HENP (n = 16)			LEFP (n = 16)			LENP (n = 16)		
		before	after	*P*	before	after	*P*	before	after	*P*	before	after	*P*
BCVA (logMAR)		0.22 ± 0.21	0.25 ± 0.14	0.638	0.11 ± 0.19	0.13 ± 0.18	0.443	0.30 ± 0.19	0.23 ± 0.18	0.045	0.15 ± 0.15	0.14 ± 0.14	0.859
IOP (mmHg)		17.06 ± 4.16	14.37 ± 2.81	0.008	17.37 ± 1.86	15.48 ± 1.84	0.023	19.70 ± 7.46	15.98 ± 3.28	0.218	17.25 ± 5.95	14.65 ± 3.18	0.147
Pupil diameter (mm)		2.39 ± 0.28	2.61 ± 0.43	0.140	2.95 ± 0.31	3.25 ± 0.40	0.088	2.68 ± 0.36	2.59 ± 0.61	0.464	2.92 ± 0.50	3.17 ± 0.52	0.076
Central anterior chamber depth (mm)		2.00 ± 0.15	2.01 ± 0.12	0.528	1.99 ± 0.18	2.00 ± 0.18	0.168	2.12 ± 0.19	2.13 ± 0.19	0.557	1.90 ± 0.29	1.85 ± 0.30	0.221
Anterior chamber volume (mm^3^)		71.20 ± 7.47	90.30 ± 9.94	0.000	80.00 ± 12.10	88.00 ± 14.24	0.050	88.90 ± 10.29	105.90 ± 12.92	0.004	76.80 ± 20.16	87.50 ± 26.90	0.012
AOD500 (µm)	12:00	72.70 ± 56.99	187.70 ± 63.58	0.001	67.00 ± 76.43	140.00 ± 94.77	0.004	110.10 ± 87.19	188.60 ± 99.51	0.005	95.10 ± 94.74	174.30 ± 84.40	0.009
	6:00	84.60 ± 84.48	270.30 ± 53.03	0.000	134.30 ± 69.67	288.60 ± 123.67	0.000	124.90 ± 67.86	244.80 ± 148.20	0.018	69.60 ± 67.86	276.40 ± 109.84	0.001
	9:00	187.30 ± 58.36	288.60 ± 68.07	0.002	146.80 ± 65.86	234.60 ± 94.42	0.014	164.20 ± 86.28	273.40 ± 50.59	0.001	108.00 ± 82.83	218.90 ± 94.40	0.001
	3:00	146.90 ± 57.82	277.90 ± 78.43	0.001	171.10 ± 67.64	245.80 ± 84.75	0.008	156.89 ± 66.11	238.33 ± 78.51	0.002	109.90 ± 75.74	214.40 ± 74.99	0.001
AOD750 (µm)	12:00	113.50 ± 44.49	242.00 ± 50.70	0.000	101.20 ± 87.86	187.70 ± 97.09	0.003	164.80 ± 122.71	268.10 ± 135.89	0.032	123.00 ± 112.65	300.90 ± 183.80	0.018
	6:00	136.00 ± 69.83	336.50 ± 70.14	0.000	166.70 ± 86.31	353.10 ± 141.59	0.000	164.10 ± 75.23	313.80 ± 97.44	0.000	109.00 ± 98.68	323.40 ± 139.96	0.003
	9:00	228.20 ± 43.42	352.90 ± 77.98	0.001	194.00 ± 64.83	298.80 ± 111.77	0.005	238.10 ± 98.00	354.80 ± 93.61	0.024	160.60 ± 119.79	304.60 ± 125.91	0.000
	3:00	235.50 ± 191.43	420.50 ± 197.68	0.046	205.20 ± 89.49	311.90 ± 96.07	0.007	223.78 ± 62.75	306.56 ± 80.33	0.018	145.00 ± 120.26	306.20 ± 187.60	0.018
TIA500 (degree)	12:00	8.32 ± 6.48	20.42 ± 6.03	0.001	7.66 ± 8.59	15.27 ± 9.63	0.003	12.60 ± 9.71	20.20 ± 9.11	0.002	10.46 ± 10.30	19.00 ± 8.46	0.005
	6:00	10.03 ± 9.49	28.50 ± 5.43	0.000	14.94 ± 7.19	29.43 ± 10.78	0.000	14.60 ± 7.09	25.10 ± 11.89	0.009	8.06 ± 7.99	28.81 ± 8.65	0.001
	9:00	20.41 ± 5.70	29.82 ± 6.29	0.002	16.39 ± 6.37	24.45 ± 9.21	0.017	17.50 ± 8.03	28.30 ± 5.19	0.000	11.96 ± 9.35	22.89 ± 9.69	0.001
	3:00	16.53 ± 6.17	28.99 ± 6.85	0.001	18.29 ± 7.01	26.45 ± 8.46	0.007	17.33 ± 6.08	26.67 ± 6.76	0.001	12.42 ± 8.07	23.07 ± 6.77	0.001
TIA750 (degree)	12:00	8.38 ± 3.16	18.10 ± 3.88	0.000	7.64 ± 6.57	14.07 ± 7.02	0.003	12.20 ± 8.55	20.00 ± 8.54	0.012	9.35 ± 7.83	18.62 ± 6.30	0.001
	6:00	10.13 ± 5.04	24.43 ± 4.45	0.000	12.64 ± 6.41	24.90 ± 8.71	0.000	13.00 ± 5.74	23.90 ± 6.05	0.000	8.83 ± 7.96	24.05 ± 8.71	0.002
	9:00	16.88 ± 3.13	25.50 ± 4.73	0.001	14.51 ± 4.14	21.72 ± 7.21	0.003	17.90 ± 6.71	25.80 ± 6.07	0.021	11.77 ± 8.44	22.62 ± 8.09	0.000
	3:00	13.47 ± 4.69	23.61 ± 5.97	0.001	15.32 ± 6.00	22.64 ± 6.63	0.006	16.89 ± 4.37	22.44 ± 5.15	0.026	11.30 ± 8.45	19.69 ± 7.04	0.001

AOD: anterior opening distance; BCVA: best-corrected visual acuity; IOP: intraocular pressure; TIA: trabecular iris angle;HEFP: high energy, far from the periphery; HENP: high energy, near the periphery; LEFP: low energy, far from the periphery; LENP: low energy, near the periphery

**Fig. 6:**
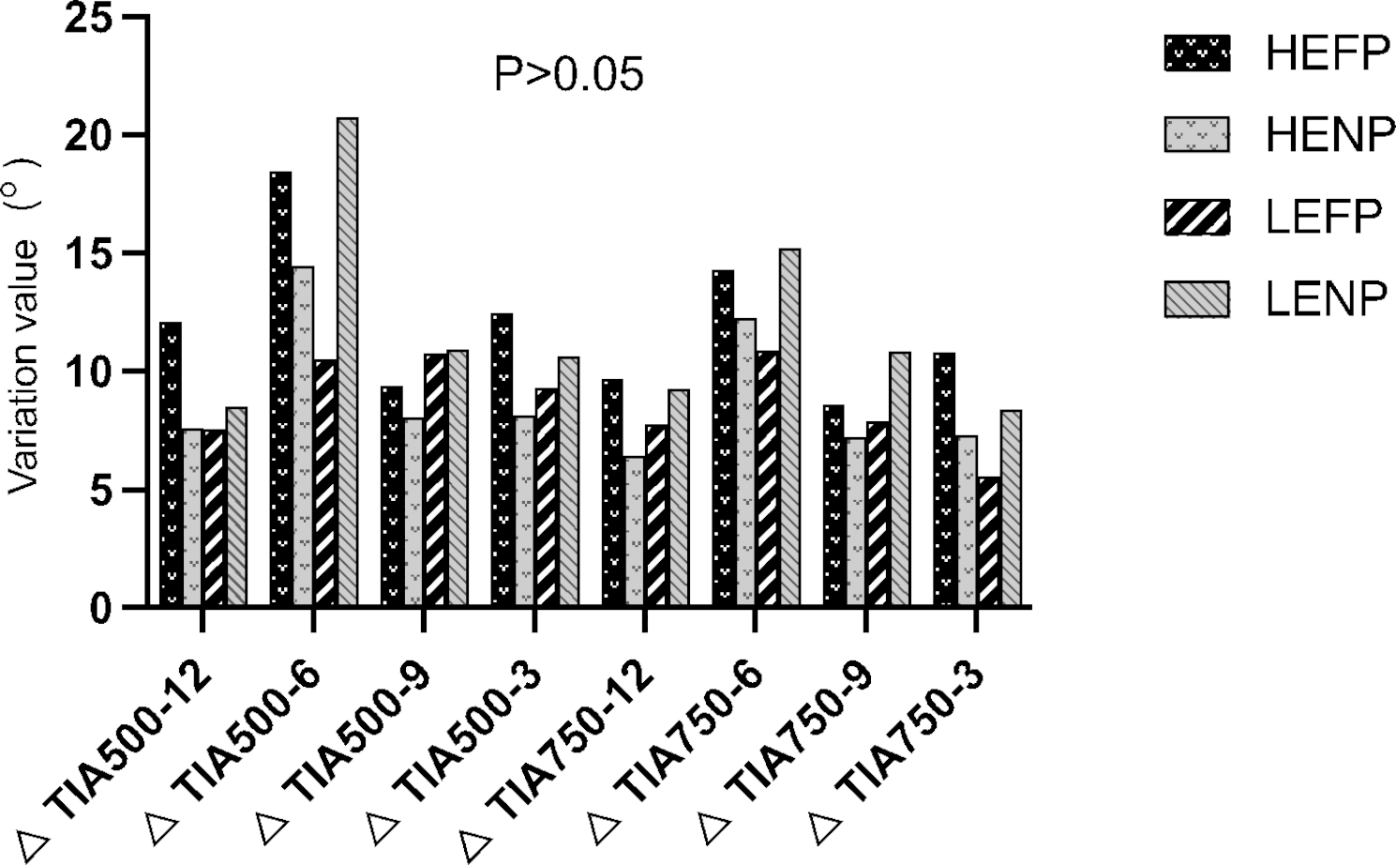
Changes in trabecular iris angle (TIA) among groups after treatment

**Fig. 7:**
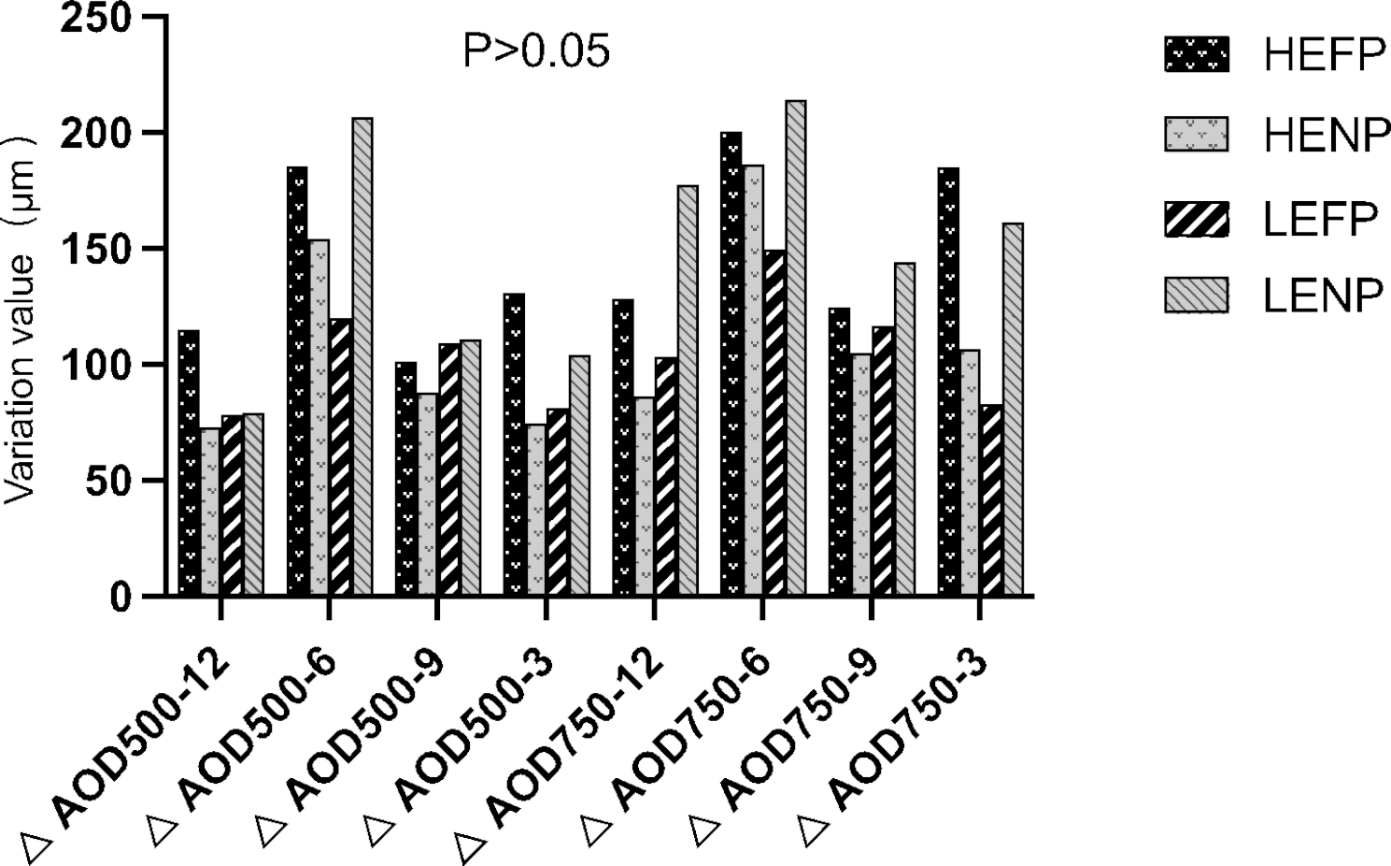
Changes in anterior opening distance (AOD) among groups after treatment

At each time point, no statistically significant difference in IOP, pupil diameter, central anterior chamber depth, anterior chamber volume, AOD500, AOD750, TIA500, and TIA750 was found among the four study groups. However, when comparing two groups, HEFP had a significantly stronger pupil dilation effect than LENP (*P* = 0.045); The anterior chamber volume in HENP was significantly larger than that in HEFP( *P* = 0.038). The change in TIA500 at 6:00 in LENP was significantly smaller than that in LEFP (*P* = 0.038). No further significant difference was found among the other parameters (Table 4; Figs. 6 and 7).

**Table 4:** Changes in parameters after treatment

Variable		HEFP (n = 16)	HENP (n = 16)	LEFP (n = 16)	LENP (n = 16)	F	*P*
△IOP (mmHg)		2.69 ± 2.51	1.89 ± 2.18	3.72 ± 8.87	2.60 ± 5.17	0.195	0.899
△Pupil diameter (mm)		0.221 ± 0.43	0.31 ± 0.51	-0.09 ± 0.38	0.25 ± 0.38	1.732	0.178
△Central anterior chamber depth (mm)		0.01 ± 0.04	0.01 ± 0.03	0.01 ± 0.06	-0.05 ± 0.12	1.783	0.168
△Anterior chamber volume (mm^3^)		19.10 ± 7.31	8.00 ± 11.17	17.00 ± 14.20	10.70 ± 10.85	2.185	0.107
△AOD500 (µm)	12:00	115.00 ± 72.91	73.00 ± 61.18	78.50 ± 66.24	79.20 ± 74.74	0.779	0.514
	6:00	185.70 ± 101.96	154.30 ± 90.23	119.90 ± 131.09	206.80 ± 143.60	1.021	0.395
	9:00	101.30 ± 75.66	87.80 ± 91.17	109.20 ± 73.78	110.90 ± 74.04	0.178	0.911
	3:00	131.00 ± 88.19	74.70 ± 70.47	81.44 ± 55.30	104.50 ± 67.23	1.246	0.308
△AOD750 (µm)	12:00	128.50 ± 67.13	86.50 ± 67.17	103.30 ± 128.42	177.90 ± 194.89	1.000	0.404
	6:00	200.50 ± 113.10	186.40 ± 99.78	149.70 ± 88.38	214.40 ± 167.94	0.527	0.667
	9:00	124.70 ± 80.61	104.80 ± 89.55	116.70 ± 136.04	144.00 ± 67.38	0.289	0.833
	3:00	185.00 ± 252.49	106.70 ± 95.83	82.78 ± 84.17	161.20 ± 175.66	0.759	0.525
△TIA500 (degree)	12:00	12.10 ± 7.50	7.61 ± 6.10	7.60 ± 5.66	8.54 ± 7.23	1.028	0.392
	6:00	18.47 ± 10.79	14.49 ± 8.06	10.50 ± 9.99	20.75 ± 13.10	1.802	0.164
	9:00	9.41 ± 7.08	8.06 ± 8.67	10.80 ± 5.37	10.93 ± 6.89	0.361	0.782
	3:00	12.46 ± 8.07	8.16 ± 7.47	9.33 ± 5.29	10.65 ± 6.83	0.684	0.568
△TIA750 (degree)	12:00	9.72 ± 4.97	6.43 ± 4.90	7.80 ± 7.80	9.27 ± 6.06	0.611	0.612
	6:00	14.30 ± 7.58	12.26 ± 6.62	10.90 ± 5.47	15.22 ± 11.25	0.593	0.624
	9:00	8.62 ± 5.18	7.21 ± 5.73	7.90 ± 8.99	10.85 ± 4.98	0.603	0.617
	3:00	10.82 ± 5.62	7.32 ± 6.41	5.56 ± 6.13	8.39 ± 5.57	1.316	0.285

AOD: anterior opening distance; IOP: intraocular pressure; TIA: trabecular iris angle;HEFP: high energy, far from the periphery; HENP: high energy, near the periphery; LEFP: low energy, far from the periphery; LENP: low energy, near the periphery

## Discussion

### Treatment effect of LPI combined with LPIp

In PACD, the iris blocks the channel draining aqueous humour, thereby causing acute or chronic IOP increases. Previous PACD treatment mainly comprised LPI, which uses a laser to establish a new drainage channel in the iris to relieve the blockage. However, it was discovered in the Zhongshan Angle-Closure Prevention Trial that eyes with suspected PAC had residual angle closure after single iridotomy and the greater angle width following iridotomy further decreased as time went on.[9] Liwan eye study also found that iridotomy alone does not improve aqueous humour drainage in more than one-third of all patients. They need additionally LPIp, and LPI combined with LPIp can more effectively relieve peripheral anterior adhesions than LPI alone [4].

PACS is considered a risk factor for PAC and PACG. Though in Handan eye study only lower than 10% of PACS found by health examination developed into PAC or PACG, logistic regression analysis identified narrower mean angle width to be associated with the progression. [10] Our study also included patients with PACS. The patients we treated were all outpatients in glaucoma clinic with plateaued or hypertrophic peripheral iris. They have the narrower angle and higher risk of progression during the further period than healthy population.

Therefore, in this study, we used LPI combined with LPIp to treat PACS and PAC in patients with plateau iris or hypertrophic peripheral iris.

LPIp was first proposed in the 1970s [11], but the type of laser used at that time had strong thermal effects causing damage. After continuous improvement, researchers tried to treat PACG with various light sources including argon, krypton, and diode lasers. At present, argon lasers are mainly used for treatment [12–14]. During LPIp, it is necessary to guide 1–2 light spots at the root of the iris using contact lenses [15]. The size of the laser light spot is 150 to 500 μm, the energy varies from 100 to 300 mW, and the exposure time ranges from 0.4 to 0.5 s [16]. LPIp causes contraction of the peripheral iris matrix thereby physically pulling the trabecular meshwork to open the chamber angle. Such traction is effective for adhesive closures and fresh peripheral anterior adhesions [17, 18]. Histopathological studies found that laser light energy, which is absorbed by iris melanocytes and collagen around blood vessels, dissipates heat to cause thermal damage. Then, fibroblasts proliferate at the site of the thermal damage, resulting in further contraction of the cell membrane [14]. As the matrix in the laser-irradiated iris area shrinks, the iris becomes thinner, which increases the distance between the iris and trabecular meshwork, thereby reducing the probability of angle closure caused by peripheral iris accumulation [19]. This suggests that LPI mainly relieves pupil block, but LPIp is helpful to resolve chronic angle closure caused by other mechanisms including plateau iris [17, 20].

After LPI combined with LPIp treatment, the IOP of all enrolled patients was significantly decreased compared to that before surgery. The results regarding the IOP-lowering effects of LPIp differ among published studies. Lee et al. found that the IOP values of patients treated with either LPI alone or LPI combined with LPIp were not significantly decreased at 1 h, 1 day, 1 week, 1 month, and 3 months after surgery [21], but this was related to low preoperative IOP values of the enrolled patients. A study with high IOP values at baseline confirmed that both LPI alone and LPI combined with LPIp significantly reduced IOP at 12 months after surgery [22]. The current study also compared the IOP within each group before and after surgery and found that the IOP in the high-energy group was significantly decreased after surgery, whereas the IOP in the low-energy group was not. This may be due to the thermal contraction of the iris generated by laser energy, which broadens the narrow or occluded angle and can at the same time pull the scleral spur backwards. The Schlemm channel is widened from a fissure to a circular shape, generating negative pressure in the Schlemm channel and redistributing aqueous humour from the anterior chamber to the Schlemm channel. The iris contraction can also affect the trabecular meshwork, widening its gap, enlarging the mesh, and facilitating the outflow of aqueous humour. Higher energy can generate greater traction force on the trabecular meshwork, make the mesh larger, and reduce the resistance for aqueous humour outflow similar to the mechanism by which cataract surgery and parasympathetic drugs reduce the IOP [23, 24]. However, this difference is not obvious when comparing IOP reductions in each group. Some studies believe that although an IOP reduction during daytime may not be obvious after LPIp treatment, it can significantly reduce the positive rates in dark-room and prone tests, indicating its significance to maintain a stable IOP at night [12]. Therefore, in the future, we should also observe 24-h IOP fluctuations before and after surgery to evaluate the effectiveness of LPIp with different locations and energy levels more comprehensively.

Our study results showed that after LPI combined with LPIp, the central anterior chamber depth was not significantly increased, whereas the peripheral AOD, TIA, and anterior chamber volume were. Numerous studies have shown that LPI alone or combined with LPIp can deepen the central anterior chamber [21]. Consistent with our current results, the study by Bourne et al. also confirmed that after LPIp, the AOD 500/750 and TIA 500/750 values were increased [25]. It is generally believed that aqueous humour can directly flow from the posterior chamber to the anterior chamber after LPI for PAC or PACS in patients with pupil block as the main pathogenic factor. Thus, reducing the pressure difference between the anterior and posterior chambers can flatten the bulging iris and increase the depth of the central anterior chamber [26]. Our analysis demonstrated that the reason for this difference is that all study groups comprise patients with plateau iris or hypertrophic peripheral iris which is different from the inclusion criteria of previous studies. This can explain why among our study groups, the depth of the central anterior chamber is not significantly increased after laser treatment, whereas the peripheral anterior chamber is deepened, the chamber angle is increased, and the volume of the anterior chamber is significantly increased.

However, when comparing every two groups, the anterior chamber volume of HENP was larger than that of HEFP. This indicates that the higher energy is and the closer the laser point is to the periphery, the stronger the effect is on the increase in anterior chamber volume. When the laser energy is low, the selection of the spot position does not affect the anterior chamber volume. At present, clinical research has not reported on this phenomenon, but an animal experiment came to the same conclusion as the current study [27]. This might be due to the laser-induced thermal contraction of the iris widening the narrow or occluded angle. The higher the laser energy, the more significant the effect of the spot position on the efficacy, i.e., the closer the laser spot is to the periphery, the stronger the force of the facula contraction on the opening of the chamber angle (but not the force of the pupil opening).

### Safety of LPIp combined with LPI

The reported complications of LPI combined with LPIp include hyphema in the anterior chamber, transiently increased IOP, decreased corneal endothelial cell count, decreased vision, transient atelectasis pupil, corneal endothelial cell burn, persistent uveitis, and malignant glaucoma. The incidence of various complications differs among studies. Lee et al. [21] reported that 4% of patients had anterior chamber haemorrhage, whereas Sun et al. [22] reported that iris haemorrhage was observed in 12.3% and 11.7% of patients with LPI alone and LPI combined with LPIp, respectively. Another common complication is transient ocular hypertension. Sun et al. found that in the LPI group, IOP was transiently increased in 16.9% of patients, and this percentage was even higher in the LPI plus LPIp group with 17.3%. The study by Lee et al. showed that transiently increased IOP was as high as 33%[21]. However, the definitions of transiently increased IOP were different between the two studies. The former defined that the IOP exceeded 30 mmHg, whereas the latter defined transiently elevated IOP as an increase by more than 5 mmHg compared to the preoperative value. In our study, none of these serious complications occurred. We found that the pupil diameter in each group was not significantly changed after surgery compared with the value before surgery. Only HEFP had a stronger effect on pupil diameter increase than group LENP. This suggests that if LPIp is carried out with high laser energy, the laser spot should be close to the periphery to avoid an increase in pupil diameter and the accompanying photophobia or other discomforts.

### Comparison of the anterior chamber angle morphology

We can directly observe the structure of the anterior chamber angle through gonioscopy, which is regarded as the gold standard for the evaluation of this angle. However, this examination is subjective, requires from the examiner some experience, and cannot accurately quantify the angle. Furthermore, the lens must have contact with the cornea, which is difficult to achieve for patients who are at risk of infection or unable to cooperate. UBM can provide more accurate images of both anterior and posterior chamber morphology, but patients need to be examined in a supine position. The eye cup needs to be placed in the conjunctival sac, which increases the infection risk and decreases comfort during the examination [28, 29]. The Pentacam is a good tool for anterior segment imaging and quantitative measurements. It can quickly calculate anterior chamber depth, pupil diameter, anterior chamber volume, and other parameters, but the accuracy of anterior chamber angle calculation is not good in patients with plateau iris [30]. AS-OCT is a fast and noncontact method for imaging structures of the anterior chamber angle. The sensitivity of AS-OCT for detecting angle closure is higher than that of gonioscopy, leading to a higher detection rate than gonioscopy [31].

Our research has some limitations. First, the sample size of this study is small, we will further conduct a large sample multicenter study to obtain more meaningful results. Second, we did not conduct dynamic follow-ups of the enrolled patients. In future research, we should conduct dynamic follow-ups. In addition, at the initial stage of enrolment, the patients were randomly enrolled, and the baseline conditions of the patients were not matched, so the baseline levels slightly differed among groups. However, as we also compared pre- and postoperative changes within each group, the research results still have credibility. In the future, we will match the baseline conditions of patients to obtain more accurate results.

In summary, LPI combined with LPIp can effectively reduce IOP, increase anterior chamber volume, increase AOD, and widen TIA. Intraoperatively, high-energy laser spots can obtain the best effect and safety when located one spot diameter from the scleral process. Swept-source AS-OCT can safely and effectively quantify the structure of the anterior chamber angle.

## Data Availability

The data used to support the findings of this study are available from the corresponding author upon request.

## List of abbreviations

AOD: anterior opening distance
AS-OCT: anterior segment optical coherence tomography
BCVA: best-corrected visual acuity
IOP: intraocular pressure
LPI: laser peripheral iridotomy
LPIp: laser peripheral iridoplasty
OCT: optical coherence tomography
PACD: primary angle closure disease
PAC: primary angle closure
PACG: primary angle closure glaucoma
PACS: suspected primary angle closure
PAS: peripheral anterior synechiae
TIA: trabecular iris angle
UBM: ultrasound biomicroscopy
HEFP: high energy, far from the periphery
HENP: high energy, near the periphery
LEFP: low energy, far from the periphery
LENP: low energy, near the periphery
